# Outbreaks of Foot-and-Mouth Disease in Burundi, East Africa, in 2016, Caused by Different Serotypes

**DOI:** 10.3390/v14051077

**Published:** 2022-05-17

**Authors:** Andrea Isabel Estevez Garcia, David J. Lefebvre, Lionel Nyabongo, Andy Haegeman, Canesius Nkundwanayo, Annebel De Vleeschauwer, Désiré Ntakirutimana, Ilse De Leeuw, Deogratias Nsanganiyumwami, Pascal Niyokwizera, Thierry van den Berg, Alfred Niyokwishimira, Kris De Clercq

**Affiliations:** 1 Unit for Exotic Viruses and Particular Diseases, Department of Infectious Diseases in Animals, Sciensano, 1180 Brussels, Belgium; andreagarcia@fam.edu.br (A.I.E.G.); andy.haegeman@sciensano.be (A.H.); annebel.devleeschauwer@ugent.be (A.D.V.); ilse.deleeuw@sciensano.be (I.D.L.); thvanne@gmail.com (T.v.d.B.); kris.declercq@sciensano.be (K.D.C.); 2 Faculty of Veterinary Medicine, Americana College, Americana 13477-360, Brazil; 3 Unit of Virology, Laboratoire National Vétérinaire (LNV), Bujumbura P.O. Box 227, Burundi; l.nyabongo@cgiar.org (L.N.); cnkundwanayo@yahoo.fr (C.N.); niyokwizerapascal@yahoo.fr (P.N.); alfred.niyokwishimira@isabu.bi (A.N.); 4 Impact at Scale, International Livestock Research Institute (ILRI), Bujumbura P.O. Box 1893, Burundi; 5 Faculty of Veterinary Medicine, Ghent University, 9820 Merelbeke, Belgium; 6 Direction de la Santé Animale (DSA), Gitega P.O. Box 161, Burundi; desirentak@gmail.com (D.N.); nsanga5@yahoo.fr (D.N.); 7 Ministère des Infrastructures, de l’Equipement et des Logements Sociaux, Bujumbura P.O. Box 1860, Burundi; 8 Institut des Sciences Agronomiques du Burundi (ISABU), Bujumbura P.O. Box 795, Burundi

**Keywords:** foot-and-mouth disease, FMD, virus, FMDV, Burundi, serotype A, serotype SAT2, serology, phylogeny, VP1

## Abstract

Burundi is a small, densely populated country in the African Great Lakes region. In March 2016, several hundreds of cattle were reported with vesicular lesions, suggesting foot-and-mouth disease (FMD). Epithelial samples, saliva, and blood were collected in six of the affected provinces spread over the country. The overall seroprevalence of FMD virus (FMDV) in the affected herds, as determined by antibodies against FMDV non-structural proteins, was estimated at 87%. Antibodies against FMDV serotypes O (52%), A (44%), C (19%), SAT1 (36%), SAT2 (58%), and SAT3 (23%) were detected across the provinces. FMDV genome was detected in samples from five of the six provinces using rRT-PCR. FMDV was isolated from samples from three provinces: in Cibitoke province, serotypes A and SAT2 were isolated, while in Mwaro and Rutana provinces, only serotype SAT2 was isolated. In Bururi and Cankuzo provinces, the serological profile suggested a recent incursion with serotype SAT2, while in Bubanza province, the serological profile suggested past incursions with serotype O and possibly serotype SAT1. The phylogenetic assessments showed the presence of topotypes A/Africa/G-I and SAT2/IV, similarly to previously characterized virus strains from other countries in the region, suggesting a transboundary origin and necessitating a regional approach for vaccination and control of FMD.

## 1. Introduction

Foot-and-mouth disease (FMD) is highly contagious and causes severe economic losses in susceptible cloven-hoofed animals, including cattle, sheep and goats, swine, and many wildlife species [1]. The etiological agent is a virus from the *Aphthovirus* genus, *Picornaviridae* family, and causes vesicular lesions on feet, oral mucosa, and mammary glands that cannot be differentiated clinically from other vesicular diseases [2]. There are seven antigenic groups or serotypes of FMD virus (FMDV): O, A, C, SAT1, SAT2, SAT3, and Asia1, and although there is no cross-protection among serotypes [2], there is considerable serological cross-reaction [3,4,5,6]. The genetic variation among FMDV serotypes evidences the independent evolution and circulation of viral strains in different genotypic groups, so-called pools. There are seven pools described [7] and Burundi, where the current study was conducted, belongs to pool 4 (Eastern Africa), with FMDV circulating serotypes O, A, SAT1, SAT2, and SAT3 [8], and is situated at the border with neighboring pool 5 (Western/Central Africa). FMD is endemic in most parts of Africa and the epidemiological situation is complex due to marked differences in the geographic distribution of serotypes, simultaneous presence of different serotypes and topotypes in the same region, high intratypic variation, and the presence of wildlife that can act as a reservoir [9,10,11]. In the ten-year period before the sampling year of this study (2016), multiple topotypes from FMDV serotypes O, A, SAT1, SAT2, and SAT3 were reported in Africa [12], with the last report of serotype C in 2004 [13,14]. Most of Africa’s livestock are kept under extensive systems in arid and semi-arid lands and by smallholders in subsistence-oriented mixed crop–livestock systems [15]. In this region, livestock stimulate income flow, creating a cash reserve for paying for seeds and food during critical periods of the growing season [16]. Cattle are also a principal form of capital accumulation and are sold when larger expenses, such as school fees or medical costs, need to be covered [17]. In endemic low-income settings, FMD has a substantial impact on the food security and livelihood of the affected communities due to the direct losses, including compromised milk and meat production, as well as indirect losses caused by the disease [18].

Burundi is a small, very densely populated country with almost 12 million inhabitants in East Africa, bordering Rwanda in the north, Tanzania in the east and south, and Lake Tanganyika and the Democratic Republic of Congo (DRC) in the West (Wikipedia). More than 90% of its population depends on agriculture [19]. According to FAO, Burundi has approximately 500,000 cattle, 2 million small ruminants, and 200,000 pigs [20]. Most of its livestock are kept by smallholders in subsistence-oriented mixed crop–livestock systems, although (semi-)nomadism and transhumance on seasonal cycles occur [19]. FMD is endemic in Burundi, with inadequate surveillance and underreporting of cases. Reported FMD outbreaks are usually not further characterized due to unwillingness of farmers to pay for diagnosis as well as limited staff and laboratory capacity [21]. Although the animal health authorities advise farmers of a yearly preventive vaccination with a tetravalent FMD vaccine (serotypes O, A, SAT1, and SAT2), the degree of vaccination is very low in some communities due to the high cost of the vaccine [22].

The latest reported outbreaks of FMD in Burundi before this study were in 2012 and 2015 without information regarding the serotype involved [23], highlighting the necessity of following up FMD outbreaks [21]. In the absence of specific reports, it was assumed that FMDV was circulating in Burundi, and in March 2016 clinical signs of FMD were reported in cattle in several Burundian provinces. According to local animal health authorities, the outbreak was probably introduced by cattle coming from Tanzania, and 423 cows were reported as affected in the Bururi province [22]. There were no records of previously vaccinated cattle and the total number of animals affected in other provinces remained unknown. Control measures used to contain the outbreak consisted of closing cattle markets, banning transhumance and free-range grazing, and building a quarantine center in the province of Cankuzo at the Tanzanian–Burundi border [22,24]. The lack of information regarding the circulating FMDV serotypes in sub-Saharan Africa and the deficiency in diagnostic capacity make the implementation of surveillance and control programs difficult [25].

Therefore, the aim of this study was to provide information on the circulating FMDV strains involved in the FMD epidemic in Burundi occurring in March 2016 by means of serological, virological, and molecular investigations. This knowledge will support the implementation of science-based surveillance and control programs.

## 2. Materials and Methods

### 2.1. Samples

**Figure 1:**
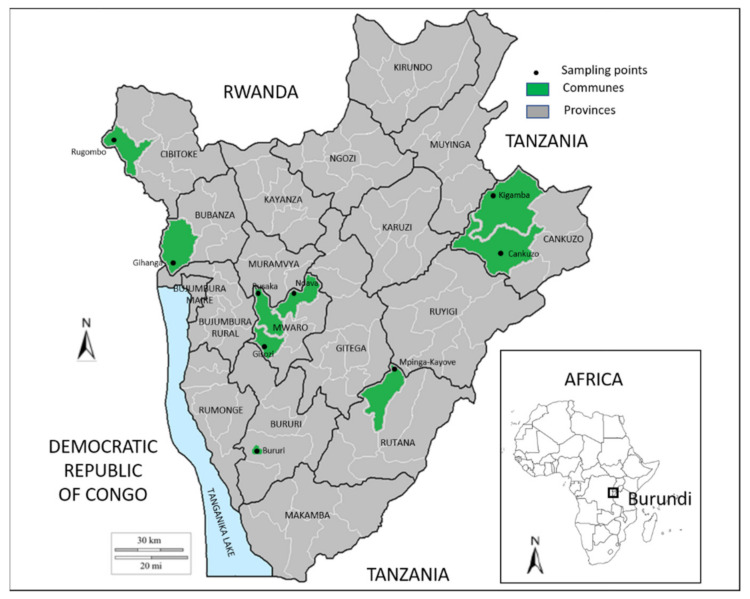
Sampling points in nine different communities spread over six provinces of Burundi (Bubanza, Bururi, Cankuzo, Cibitoke, Mwaro, and Rutana).

During the FMD outbreak starting in March 2016, samples were collected in herds with cattle presenting clinical signs compatible with FMD or with a recent history of FMD. A total of 193 epithelial and/or saliva samples and 172 blood samples were taken and a summary is presented in Table 1. Epithelium from vesicular lesions was the preferred choice. In the case of cicatrization, saliva was chosen. Epithelial and saliva samples were placed in transport medium as prescribed by the OIE [2]. The sampling points were located in nine different communities spread over six of the affected provinces as indicated in Figure 1. The population studied included animals of ages between six months and nine years old. The samples were transported to the Laboratoire National Vétérinaire (LNV) in Bujumbura, Burundi, where serum was separated and stored at −20 °C. Tissue and saliva samples were stored at −70 °C. Import and export permits were obtained from relevant authorities and samples were shipped on dry ice to Sciensano in Belgium, in order to perform the analyses as part of a capacity-building collaboration program between Sciensano (Belgium) and the LNV (Burundi).

### 2.2. Serological Tests

#### 2.2.1. Non-Structural Proteins (NSP) Antibody ELISA

Antibodies against FMDV non-structural proteins 3ABC (NSP-3ABC), differentiating animals infected with FMDV from non-infected, vaccinated animals [26], were detected by means of the commercial solid-phase competition ELISA ID Screen^®^ FMD NSP Competition (Innovative Diagnostics, previously ID.Vet, Grabels, France), according to the manufacturer’s instructions.

#### 2.2.2. Structural Proteins (SP) Antibody ELISA

In order to detect antibodies against FMDV structural proteins (SP) of serotype O, the commercial kit ID Screen^®^ FMD Type O Competition (Innovative Diagnostics, Grabels, France) was used, according to the manufacturer’s instructions. The detection of antibodies against FMDV SP of serotypes A, C, SAT1, and SAT3 was carried out by an in-house solid-phase competition ELISA (SPCE) as described previously [27]. Antibodies against FMDV SAT2 SP were detected by an SPCE kit, according to the manufacturer’s instructions (IZSLER, Brescia, Italy—The Pirbright Institute, Pirbright, UK).

### 2.3. Virus Identification and Characterization

#### 2.3.1. Real-Time RT-PCR (rRT-PCR)

The RNA extraction from tissue samples was carried out after mechanical disruption using a TissueLyser (Qiagen, Venlo, The Netherlands) and lysis with guanidinium thiocyanate-phenol-chloroform followed by RNA purification with silica column (NucleoSpin^®^ RNA Virus, Macherey-Nagel, Düren, Germany). Saliva samples were treated with the same lysis buffer before the RNA purification step with the silica column. The RNA was used in a triplex one-step rRT-PCR protocol with beta-actin as an internal control (IC) and synthetic RNA as an external control (EC), as adapted from the reference methods described in the OIE Manual [2] and described previously [28], being able to detect all the FMDV serotypes. Cp values below 40 were considered as positive.

#### 2.3.2. Virus Isolation and Antigen ELISA

All samples with rRT-PCR Cp values below 34 were submitted to virus isolation (VI) on the porcine kidney cell line IB-RS-2 as detailed before [29]. Samples yielding negative results after three consecutive passages were then submitted to VI on the ovine epithelial cell line OA3.Ts following the same protocol. In case of a positive result, as determined by the formation of cytopathic effect (CPE), the FMD virus present in the cell culture supernatant was serotyped by an in-house antigen ELISA detecting all seven serotypes of FMDV, as described in the OIE Manual [2] and as detailed previously [29].

#### 2.3.3. Conventional RT-PCR, Cloning, and Sequencing

A reverse-transcription reaction, followed by PCR, was performed on epithelial tissue and saliva. The serotyping results of the antigen ELISA guided the selection of specific primers targeting the VP1 gene of FMDV serotype A or SAT2 [30]. When there was no amplification with the serotype-specific primers, universal primers targeting VP1 were used [30]. PCR products were resolved by electrophoresis in 1% agarose gel, colored with SYBR™ Safe DNA Gel Stain (Thermo Fisher Scientific, Merelbeke, Belgium), and visualized using a UV transilluminator. PCR products were then excised with a sterile blade and purified using the QIAquick Gel Extraction Kit^®^ (Qiagen Ltd., Crawley, West Sussex, UK), according to the manufacturer’s instructions.

The purified PCR products were cloned into the pCR2.1-Topo vector using the TOPO^®^ TA^®^ kit (Invitrogen by Thermo Fisher Scientific, Merelbeke, Belgium). The transformed bacteria with the plasmid containing the PCR product of interest were selected using X-gal-containing ampicillin (50 µg/mL) LB plates. Finally, the plasmids were purified using a QIAprep Spin Miniprep Kit (Qiagen, Venlo, The Netherlands), according to the manufacturer’s instructions. The presence of the genomic region of interest was verified by *Eco* RI restriction enzyme digestion and electrophoresis in 1% agarose gel. 

The inserts of the selected clones were sequenced with M13 primers as detailed previously [29], using a commercial kit (BrightDye Terminator Cycle sequencing Kit^®^, Nimagen, Nijmegen, The Netherlands). The sequencing reactions were purified using the BigDye XTerminator™ Purification Kit (Applied Biosystems, Carlsbad, CA, USA) and run on an ABI 3130 Genetic Analyzer (Life Technologies, Gent, Belgium). The electropherograms corresponding to the sequences were visualized and edited using CHROMAS 2.6.4 (Technelysium, South Brisbane, Australia). The primer and plasmid sequences were withdrawn and a consensus sequence from forward and reverse strands was obtained using GeneDoc 2.6 Software (NRBSC, Pittsburgh, PA, USA). The polarity and identity of the sequences were verified using the web BLAST tool at https://blast.ncbi.nlm.nih.gov/Blast.cgi, accessed on 17 March 2022 [31].

#### 2.3.4. VP1 Phylogenetic Analysis

The phylogenetic evaluation of the obtained FMDV VP1 regions was performed after a best-fit model analysis. The resulting Bayesian information criterion (BIC) values were compared in combination with the obtained tree topologies of reference sequences in order to select the optimal phylogenetic settings across the two FMDV serotypes included in this study. The evolutionary histories were inferred by using: (1) the Maximum Likelihood method (ML) based on either the HKY-model [32] for FMDV serotype SAT2 or on the Tamura–Nei model [33] for FMDV serotype A. Both analyses were run with discrete Gamma distribution and evolutionarily invariable sites; (2) the Neighbor-Joining method (NJ) based on the Tamura–Nei model [33] with Gamma distribution to model evolutionary rate differences among sites. Bootstrap analysis (1000 replicates) was carried out for all methods [34], whereby branches corresponding to partitions reproduced in less than 50% of bootstrap replicates were collapsed. For the initial ML trees, the heuristic searches were obtained by applying the Neighbor-Joining method to a matrix of pairwise distances estimated using the Maximum Composite Likelihood (MCL) approach. All positions with less than 95% site coverage were eliminated. All calculations were performed in the MEGA X software package [35].

## 3. Results

### 3.1. Serological Analyses

#### 3.1.1. Detection of Antibodies against Non-Structural Proteins of FMDV

Antibodies against FMDV NSP were detected in all five provinces examined, as serum samples were not available from Mwaro province. Overall, 149/172 (86.6%) cattle tested positive, with the percentage positivity per province ranging from 77.6% in Bubanza, 84.8% in Cibitoke and Rutana, to 100% in Bururi and Cankuzo, respectively (Table 2).

#### 3.1.2. Detection of Antibodies against Structural Proteins of FMDV

One hundred and forty-nine cattle sera that were positive for anti-FMDV NSP antibodies were further tested in serotype-specific ELISA. Overall, the seroprevalence for each serotype was 51.7% for FMDV serotype O, 44.3% for A, 19.5% for C, 36.2% for SAT1, 58.4% for SAT2, and 22.8% for SAT3. The number of SP-positive samples per serotype per province is detailed in Table 2. These detailed results per province are discussed further, in the light of the results of the virological analyses obtained. A supplementary table (Table S1) shows the serological profile of the individual animals.

Then, the 23 sera that were negative for anti-NSP antibodies were tested. Fourteen samples tested negative for the six tested serotypes, while six samples tested positive for serotype C alone. One sample tested positive for serotype O alone, while two other samples tested positive for the combination A-SAT1-SAT2 and O-SAT2, respectively.

### 3.2. Virological Analyses

#### 3.2.1. Real-Time RT-PCR, Virus Isolation, and Antigen ELISA

**Figure 2:**
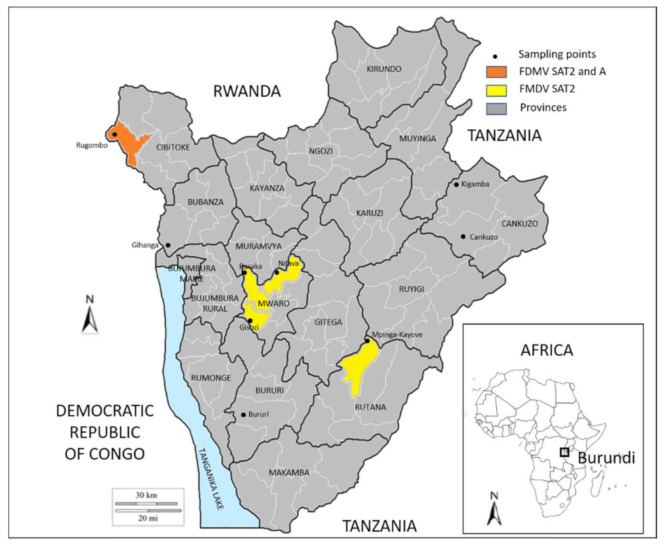
Geographical distribution and serotypes of foot-and-mouth disease virus isolates collected in Burundi in March 2016.

Seventy-nine samples were found to be positive by rRT-PCR, as shown in Table 3. Of these 79 samples, 35 had a Cp value below 34 and were submitted to virus isolation. Field virus was isolated from 18 samples and the cell supernatant was submitted to antigen ELISA. Three samples from Mwaro, nine samples from Rutana, and three samples from Cibitoke were characterized as FMDV serotype SAT2, while three other samples from Cibitoke were characterized as serotype A (Table 3 and Figure 2).

The serotype SAT2 FMDV was isolated from 15 animals, of which serum was available for 11 animals. Nine of these eleven animals tested positive for antibodies against NSP. One of these nine animals had a doubtful antibody response against SP, as the threshold for positivity was not exceeded for any of the six serotypes tested, four animals tested positive for a single serotype other than SAT2, and two animals tested positive for two or three serotypes other than SAT2. The remaining two animals tested positive for the combination A-SAT1-SAT2 and O-A-SAT2, respectively.

The serotype A FMDV from Cibitoke province was isolated from three 1-year-old animals that were negative for antibodies against NSP.

#### 3.2.2. VP1 Sequencing and Phylogenetic Analysis

The results of the different obtained FMDV sequences are summarized in Table 4.

- **Serotype A**

**Figure 3:**
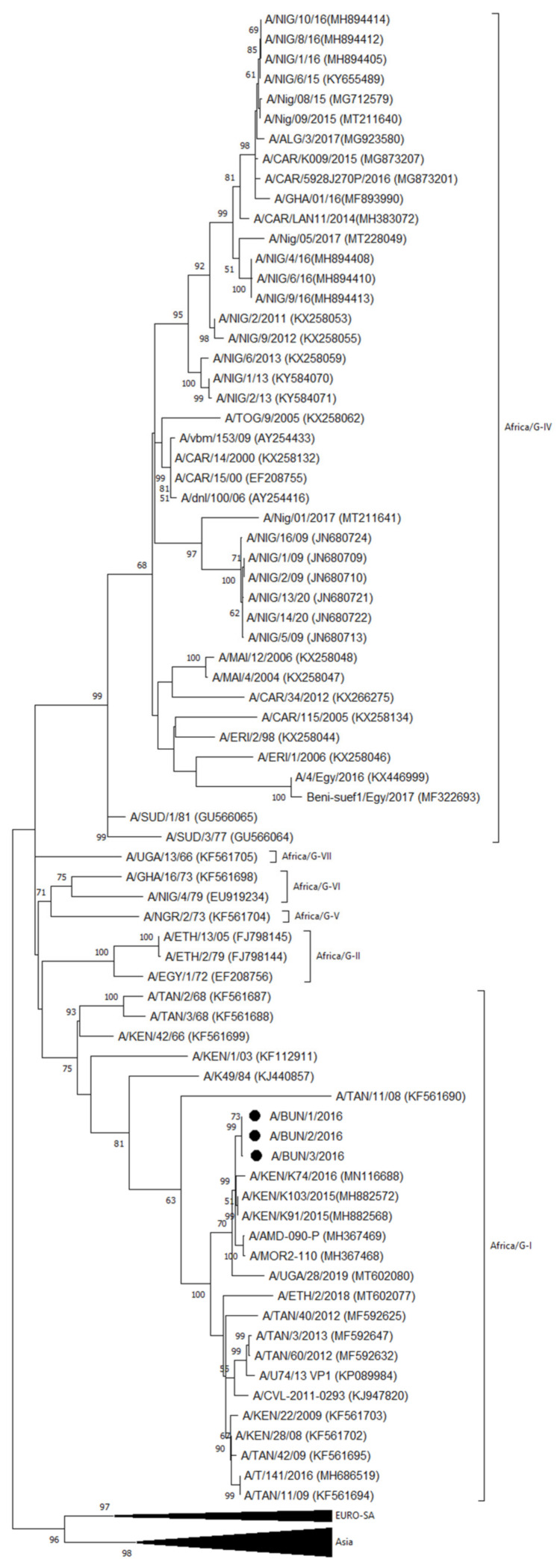
VP1 phylogenetic tree for FMDV serotype A inferred using the Maximum Likelihood method based on the Tamura–Nei model. Bootstrap values ≥ 50% are indicated at the nodes. Novel Burundian FMD virus from this study is indicated with ●.

An outbreak in Cibitoke province was characterized as FMDV serotype A by antigen ELISA and as topotype Africa lineage G-I by VP1 sequencing and phylogenetic analysis. In the NJ and ML phylogenetic trees (Figure 3), the serotype A isolates from Cibitoke province clustered with isolates from Kenya (A/KEN/K91/2015 and A/KEN/K103/2015 from 2015 and A/KEN/K74/2016 from 2016) and from Uganda (A/AMD-090-P and A/MOR2-110-P from 2016) [36,37,38], with ∼98–99% VP1 nucleotide (nt) identity with these isolates.

This cluster including the serotype A isolates from Cibitoke province branched separately from a group of predominantly Tanzanian but also Kenyan and Ugandan viruses isolated from 2008 to 2016 [39,40]. Approximately between 93 and 96% VP1 nt identity was observed between this group of viruses from Tanzania, Kenya, and Uganda, and the serotype A isolates from Burundi from the present study.

- **Serotype SAT2**

Simultaneously with the FMD serotype A outbreak in Cibitoke province, another outbreak, in the same community, notably, was characterized as FMDV serotype SAT2 by antigen ELISA and as topotype IV by VP1 sequencing and phylogenetic analysis. Similarly, the FMD SAT2 outbreaks in Mwaro and Rutana provinces were characterized as topotype IV. Between the isolates from Cibitoke and those from Mwaro and Rutana, we observed ∼98–99% VP1 nt identity, while we observed 99.7–100% VP1 nt identity between the isolates from Mwaro and Rutana.

**Figure 4:**
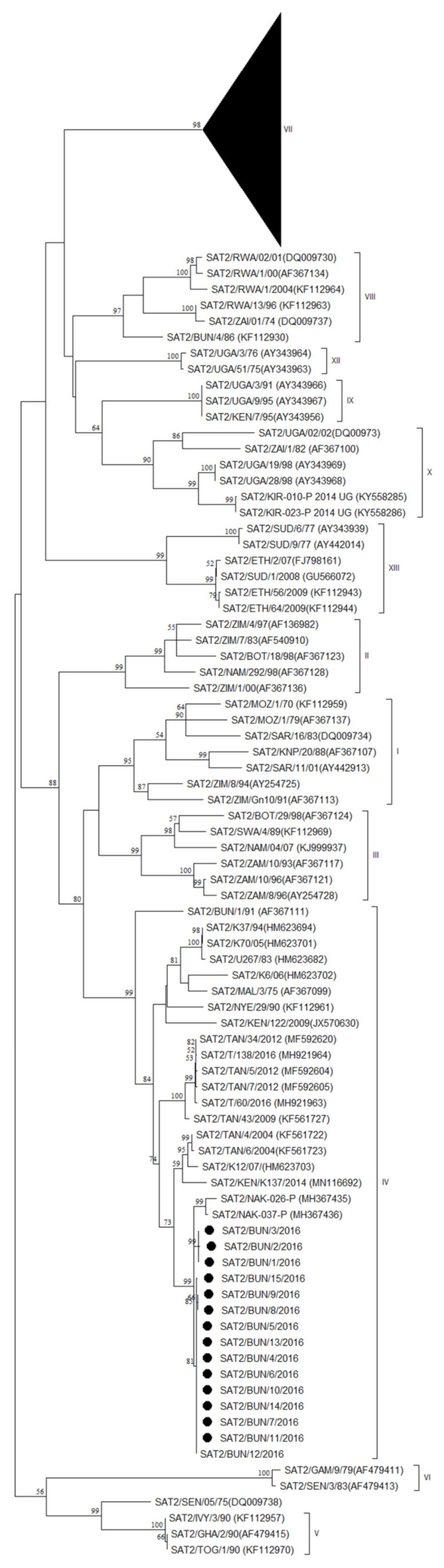
VP1 phylogenetic tree for FMDV serotype SAT2 inferred using the Maximum Likelihood method based on the HKY-model. Bootstrap values ≥ 50% are indicated at the nodes. Novel Burundian FMD virus from this study is indicated with ●.

In the NJ and ML phylogenetic trees (Figure 4), the serotype SAT2 isolates from Burundi from the present study clustered with isolates from Uganda from 2016 (SAT2/NAK-026-P and SAT2/NAK-037-P) [38] with ∼96–97% VP1 nt identity.

The isolates from Burundi from the present study branched separately from viruses isolated in Kenya and Tanzania during the period 2004 to 2016. Approximately between 90 and 91% VP1 nt identity was observed between the serotype SAT2 isolates from Burundi from the present study and a virus isolated in Kenya in September 2014 (SAT2/KEN/K137/2014) [41], and approximately between 87 and 88% VP1 nt identity was observed with viruses isolated in Tanzania during the period 2012 to 2016 (shown as SAT2/TAN/n/2012 or SAT2/T/n/2016) [40].

## 4. Discussion

In the present study, samples were collected from cases of FMD in cattle in Burundi during March 2016. The obtained FMD viruses (FMDVs) were characterized and compared with previously characterized viruses from Burundi—if available—and neighboring countries. In one community of Cibitoke province, FMDVs of the serotypes A and SAT2 were isolated, confirming previous findings that in sub-Saharan Africa different FMD virus strains can circulate at the same place at the same time [9,10,11]. The isolated viruses were of the same topotypes as contemporary FMDVs from Uganda, Kenya, and Tanzania. Interestingly, a few months before the FMD outbreaks in Burundi, highly identical serotype A viruses (∼99% VP1 nucleotide identity with the FMDV serotype A isolates from Burundi March 2016) were isolated in Kenya, A/KEN/K91/2015 in September 2015 and A/KEN/K103/2015 in October 2015. However, it is unclear how this virus strain might have been introduced into Burundi, as most livestock movement in the region, both for trade as for livestock farming, is not controlled. It is also possible that this finding simply illustrates the circulation of this virus strain in the greater region at that time and does not necessarily reflect a causal relationship. Further, Cibitoke province borders the DRC to the west and Rwanda to the north, and it should be taken into account that only very few FMDVs from the DRC and Rwanda have been genetically characterized and have sequences which are publicly available. This underreporting makes it difficult to obtain a complete overview of the circulation of FMDV in the African Great Lakes region.

While 23 out of 28 (82%) NSP-positive serum samples from Cibitoke province tested positive for FMDV serotype A, only 5 out of 28 (18%) tested positive for serotype SAT2. This suggests that, at the time of sampling, serotype SAT2 was newly introduced into Cibitoke province, while serotype A had already been circulating there for a longer time. In contrast to serotype A, serotype SAT2 was also isolated in Mwaro province and Rutana province, while in Bururi province and Cankuzo province all serum samples (100%) were positive for antibodies against FMDV NSP and serotype SAT2, suggesting that these animals were recently infected with FMDV serotype SAT2. This suggests an endemic course of serotype A infections and an epidemic course of serotype SAT2 infections in Burundi at that time. This substantiates the hypothesis of the local animal health authorities that the large nationwide outbreak of FMD in 2016 may have been introduced by infected cattle entering Burundi from Tanzania, even though the phylogenetic analysis suggests that this outbreak may have its roots in Uganda or Kenya. In this respect, the export of cattle from Uganda, through Tanzania, to Burundi can be taken into account as a potential factor to favor the circulation of FMDV in the region, as mentioned before [38]. It can then be hypothesized, based on the serological and virological profiles of the provinces, that, following the introduction of FMDV SAT2 via Tanzania into Burundi, the epidemic has spread in Burundi in a northwesterly direction. However, based on the VP1 nt sequence analysis, it can also be hypothesized that the occurrence of FMDV SAT2 in the northwestern province of Cibitoke results from a separate introduction of FMDV SAT2 into Burundi. After Cibitoke province, Bubanza province is the second most northwestern province of Burundi. In Bubanza province, the most seroprevalent FMDV serotypes were not SAT2 or A, but O (67% of the NSP-positive samples), followed by SAT1 (49% of the NSP-positive samples). This suggests previous infections with FMDV serotype O and possibly also serotype SAT1 in this province. Both these serotypes were previously observed in Burundi, serotype O in 2003 [42] and serotype SAT1 in 1999 [43]. Although the phylogenetic analyses in this study point towards Uganda, Kenya, and Tanzania as potential sources of FMDV incursions in Burundi, it should be noted that, for example, the FMDV topotype SAT2/VIII (see Figure 4 of this study) was observed in Rwanda, Burundi, and the DRC in the period 1974–2004. Taken together, it is clear that enhanced surveillance, reporting, and characterization of FMD virus is needed in Burundi. In order to improve the current FMD surveillance activities in Burundi, it can be speculated that improved education of herdsmen will enhance reporting and thus contribute to cost-effective surveillance [44]. To improve the identification of circulating FMDV strains, it may be cost-effective to enhance surveillance activities in subpopulations of cattle with a higher risk of spreading FMDV into the country, such as imported cattle at border crossings or markets [45]. Combining the surveillance for FMD with those for other major livestock diseases will reduce the total number of samples to be taken and the associated costs [46]. When rRT-PCR is available, non-invasive sampling methods such as the collection of pooled milk samples from cattle [47], pooled oral swabs from sheep, or saliva collected from pigs via ropes have the potential to be a cost-effective alternative or complementary method to traditional serological surveys [48]. With appropriate enrichment strategies, it is possible to sequence the virus when it is present in low abundance in such samples [49], illustrating the potential for sequence-based surveillance.

In Cibitoke, Mwaro, and Rutana provinces, more than three quarters (69 out of 89) of the samples tested positive for FMDV using rRT-PCR, suggesting that most animals in these provinces were sampled during the acute or subacute phase of the infection. About half (35 out of 69) of these positive samples were submitted to virus isolation. FMDV was isolated from about half (18 out of 35) of them, a success rate similar to previously reported [50]. On the one hand, this illustrates the importance of early reporting and early sampling of cases of FMD to obtain samples with a high viral load [29], but it also illustrates the challenge of sampling and transportation under tropical conditions and the negative effects of subsequent freezing, long-distance air transportation, and thawing on the success rate of isolating FMDV [51]. In Bubanza, Bururi, and Cankuzo provinces, only few samples (10 out of 104) tested positive for FMDV using rRT-PCR, while the serological profiles of most animals suggested recent infection with FMDV. One week after the appearance of lesions due to infection with FMDV, virus titers in affected tissues have already significantly declined, while lesions rapidly heal and most animals already have mounted an immune response [52]. Taking this diagnostic window into account, it seems that most animals in the provinces of Bubanza, Bururi, and Cankuzo were sampled during the convalescence phase of the infection. In all cases, it should also be taken into account that pre-existing immunity due to previous infections, or vaccination, with homologous or heterologous serotypes of FMDV may reduce virus replication and the severity of disease [53], and thus reduce the number of positive samples, the viral load in these samples, and the duration of positivity. Further, the indigenous Ankole breed represents more than 90% of the Burundian cattle herd [19], which often only develop mild or sub-clinical FMD, making clinical disease less obvious to diagnose [53]. Therefore, it is essential to combine multiple assays, as performed in this study, to maximize both the diagnostic and serotyping performances for detection and characterization of FMDV in field samples [54].

In all five provinces in which serum samples were available, the FMDV NSP seroprevalence was high, indicating a high burden of FMD in Burundi, and comparable to NSP levels found in Uganda in 2012–2013 [9]. A serological cross-reaction pattern was also observed in all five provinces, as previously observed in the region [3,4,9]. This serological cross-reaction is probably due to a combination of recent infections with FMDV, previous infections with other serotypes of FMDV, as well as cross-reaction due to some antigenic similarities among serotypes [55]. The latter seems to be the case for the serological reactions observed against serotypes C and SAT3 in this study as the last report of serotype C dates from 2004 [13,14], while SAT3 has only been isolated from cattle in East Africa on a single occasion [56]. The effect of vaccination on the serological cross-reactions observed in this study is unclear. In Burundi, a tetravalent FMD vaccine is available for serotypes O, A, SAT1, and SAT2, but the vaccination rate is low. During this study, vaccination records were not available for the sampled animals. It can be assumed that animals that have been effectively vaccinated have antibodies against at least the serotypes O, A, SAT1, and SAT2. Such a combination of antibody responses was not observed in any of the 23 animals that tested negative for antibodies against NSP. From Table S1, it can be deduced that 19 out of 149 NSP-positive animals (12.8%) had antibodies against at least the serotypes O, A, SAT1, and SAT2, whether or not in combination with antibodies against serotypes C and/or SAT3. This percentage ranged from 3.6% in Cibitoke to 21.1% in Bururi, and it is not known to what extent vaccination, infection, and serological cross-reaction each contribute to this percentage. Taken together, these data confirm the low degree of vaccination in the sampled cattle population.

The finding of FMDV SAT2/IV and A/Africa/G-I is consistent with the observed molecular epidemiology of FMDV, as the same topotypes and lineages were previously reported in other countries in the region, illustrating once more the transboundary nature of FMD [57,58]. This indicates the need for a regional approach for control and vaccination. In order to be successful, such a regional approach should be tailored to the prevailing animal husbandry and trade practices and associated transboundary animal movements [59], as movement of infected animals is the most important factor in the spread of FMD in endemically infected regions [57,58,60]. The vaccination strategy should take the multiple serotypes, topotypes, and genetic variants circulating in this part of East Africa into account [61].

In conclusion, we provided the first full documentation of outbreaks of FMD in Burundi, including serological responses, virus isolation, and antigenic and genetic virus characterization. The presence of the same virus topotypes and lineages in neighboring countries indicates a likely transboundary origin and illustrates the necessity to create a regional strategy for vaccination and control. This study also contributes to the knowledge necessary for Burundi to progress in the first stages of the Progressive Control Pathway for FMD (PCP-FMD), a risk- and evidence-based framework developed by FAO and EuFMD and endorsed by the OIE [62].

## Figures and Tables

**Table 1:** Summary of samples taken during the FMD outbreak in six provinces of Burundi, 2016.

Type of Sample	Total	Provinces					
		Bubanza	Bururi	Cankuzo	Cibitoke	Mwaro	Rutana
Saliva	86	56	18	0	6	6	0
Oral lesions *	84	0	3	10	30	9	32
Foot lesions	23	0	0	17	2	1	3
Total of tissue and/or saliva samples	193	56	21	27	38	16	35
Total of serum samples	172	58	19	29	33	0	33

* Epithelial tissue taken from vesicular lesions present in gums, oral mucosa, or tongue.

**Table 2:** Detection of antibodies against non-structural proteins (NSP) and structural proteins (SP) of FMDV in cattle after reported FMD outbreaks in Burundi in 2016.

Province	# Serum Samples Tested	# NSP Positive Samples	# SP Positive Samples per Serotype *					
			O	A	C	SAT1	SAT2	SAT3
Bubanza	58	45	30	20	9	22	15	6
Bururi	19	19	13	7	2	4	19	5
Cankuzo	29	29	17	8	0	12	29	12
Cibitoke	33	28	12	23	12	4	5	5
Rutana	33	28	5	8	6	12	19	6
Total	172	149	77	66	29	54	87	34

* Detection of antibodies against SP of FMDV in NSP-antibody-positive cattle.

**Table 3:** Diagnostic results on tissue and saliva samples from vesicular disease outbreaks in cattle in Burundi in March 2016.

Province	# Positive/Animals Tested						Serotype in Antigen ELISA	
	rRT-PCR			Virus Isolation *			A	SAT2
	saliva	oral lesion	foot lesion	saliva *	oral lesion *	foot lesion *		
Bubanza	2/56	—	—	0/0	—	—	—	—
Bururi	0/18	0/3	—	0/0	—	—	—	—
Cankuzo	—	3/10	5/17	—	0/0	0/1	—	—
Cibitoke	5/6	23/30	2/2	0/0	6/8	0/1	3/6	3/6
Mwaro	4/6	6/9	0/1	0/0	3/3	0/0	0/3	3/3
Rutana	—	26/32	3/3	—	9/19	0/3	0/9	9/9
Total	11/86	58/84	10/23	0/0	18/30	0/5	3/18	15/18
	79/193			18/35				

* Only samples with a Cp value below 34 were submitted to virus isolation.

**Table 4:** Summary of foot-and-mouth disease virus sequences from Burundi obtained in the present study.

Sample ID	Community	Province	FMDV Serotype/Topotype	GenBank Accession #
A/BUN/1/2016	Rugombo	Cibitoke	A/Africa/G-I	OM817501
A/BUN/2/2016	Rugombo	Cibitoke	A/Africa/G-I	OM817502
A/BUN/3/2016	Rugombo	Cibitoke	A/Africa/G-I	OM817503
SAT2/BUN/1/2016	Rusaka	Mwaro	SAT2/IV	OM817504
SAT2/BUN/2/2016	Rusaka	Mwaro	SAT2/IV	OM817505
SAT2/BUN/3/2016	Rusaka	Mwaro	SAT2/IV	OM817506
SAT2/BUN/4/2016	Rugombo	Cibitoke	SAT2/IV	OM817507
SAT2/BUN/5/2016	Rugombo	Cibitoke	SAT2/IV	OM817508
SAT2/BUN/6/2016	Rugombo	Cibitoke	SAT2/IV	OM817509
SAT2/BUN/7/2016	Mpinga-Kayove	Rutana	SAT2/IV	OM817510
SAT2/BUN/8/2016	Mpinga-Kayove	Rutana	SAT2/IV	OM817511
SAT2/BUN/9/2016	Mpinga-Kayove	Rutana	SAT2/IV	OM817512
SAT2/BUN/10/2016	Mpinga-Kayove	Rutana	SAT2/IV	OM817513
SAT2/BUN/11/2016	Mpinga-Kayove	Rutana	SAT2/IV	OM817514
SAT2/BUN/12/2016	Mpinga-Kayove	Rutana	SAT2/IV	OM817515
SAT2/BUN/13/2016	Mpinga-Kayove	Rutana	SAT2/IV	OM817516
SAT2/BUN/14/2016	Mpinga-Kayove	Rutana	SAT2/IV	OM817517
SAT2/BUN/15/2016	Mpinga-Kayove	Rutana	SAT2/IV	OM817518

## Data Availability

The datasets analyzed during the present study are available from the authors upon reasonable request. The nucleotide sequences obtained in the present study are accessible in GenBank under the numbers indicated in Table 4 of the manuscript.

## Supplementary Materials

The following supporting information can be downloaded at: https://www.mdpi.com/article/10.3390/v14051077/s1, Table S1: Serological profile in cattle after reported FMD outbreaks in Burundi in 2016.
