# Supplementary material for: Outbreaks of Foot-and-Mouth Disease in Burundi, East Africa, in 2016, Caused by Different Serotypes
Source: Viruses. 2022 May 17;14(5):1077. doi: 10.3390/v14051077 (PMC9143720; doi:10.3390/v14051077)
Supplement: Supplementary file 1 [file viruses-14-01077-s001.zip › Supplementary Table 1.pdf]

viruses

MDPI

Table S1. Serological profile in cattle after reported FMD outbreaks in Burundi in 2016.

**Province**	**# NSP positive samples**	**# samples positive for a particular serotype of FMDV or positive for a particular combination of serotypes**																	
		Monovalent serum							Polyvalent serum positive for at least O, A, SAT1 and SAT2				Polyvalent serum positive for other combinations of sero-types						
		O	A	C	SAT1	SAT2	SAT3	doubt ful*	O, A, SAT1 SAT2	O, A, C, SAT1 SAT2	O, A, SAT1 SAT2 SAT3	O, A, C, SAT1 SAT2 SAT3	O, A	O, A, C	O, A, SAT1	O, A, SAT2	O, A, SAT3	O, A, SAT1 SAT3	O, A, SAT2 SAT3
Bubanza	45	5	2		1	2		6	2	2	2	1	1		4	1	1		
Bururi	19					6			1		1	2				1			2
Cankuzo	29					10					5					2			
Cibitoke	28		7	1		1		3				1	2	3			1	2	
Rutana	28	1	1	2		5		4			1	1				1			
Total	149	6	10	3	1	24	0	13	3	2	9	5	3	3	4	5	2	2	2

* Doubtful result when the threshold for positivity was not exceeded for any of the six serotypes tested.

Table S1 (continued). Serological profile in cattle after reported FMD outbreaks in Burundi in 2016.

**Province**	**# samples positive for a particular serotype of FMDV or positive for a particular combination of serotypes**																		
	Polyvalent serum positive for other combinations of serotypes																		
	O, C	O, C, SAT1 SAT2	O, C, SAT1 SAT2 SAT3	O, SAT1	O, SAT2	O, SAT1 SAT2	O, SAT2 SAT3	O, SAT1 SAT2 SAT3	A, C	A, C, SAT2 SAT3	A, SAT1 SAT2	A, SAT2	A, SAT1 SAT2 SAT3	C, SAT1	C, SAT1 SAT2 SAT3	O, A, C, SAT1	O, A, C, SAT2	O, A, C, SAT1 SAT3	SAT1 SAT2	SAT1 SAT2 SAT3
Bubanza	1	1		5	1	1		1	2			1		1				1		
Bururi					6															
Cankuzo					2	1	2	5				1							1	
Cibitoke									3	1						1	2			
Rutana			1								1	2	1	1	1				4	1
Total	1	1	1	5	9	2	2	6	5	1	1	4	1	2	1	1	2	1	5	1
